# The polyglutamine protein ATXN2: from its molecular functions to its involvement in disease

**DOI:** 10.1038/s41419-024-06812-5

**Published:** 2024-06-14

**Authors:** Rafael G. Costa, André Conceição, Carlos A. Matos, Clévio Nóbrega

**Affiliations:** 1 Algarve Biomedical Center Research Institute (ABC-RI), Faro, Portugal; 2 PhD program in Biomedical Sciences, Faculdade de Medicina e Ciências Biomédicas, Universidade do Algarve (UAlg), Faro, Portugal; 3 Faculdade de Medicina e Ciências Biomédicas, Universidade do Algarve (UAlg), Faro, Portugal; 4 Center for Neuroscience and Cell Biology (CNC), Coimbra, Portugal; 5 Champalimaud Research Program, Champalimaud Center for the Unknown, Lisbon, Portugal

**Keywords:** Cellular neuroscience, Spinocerebellar ataxia

## Abstract

A CAG repeat sequence in the *ATXN2* gene encodes a polyglutamine (polyQ) tract within the ataxin-2 (ATXN2) protein, showcasing a complex landscape of functions that have been progressively unveiled over recent decades. Despite significant progresses in the field, a comprehensive overview of the mechanisms governed by ATXN2 remains elusive. This multifaceted protein emerges as a key player in RNA metabolism, stress granules dynamics, endocytosis, calcium signaling, and the regulation of the circadian rhythm. The CAG overexpansion within the *ATXN2* gene produces a protein with an extended poly(Q) tract, inducing consequential alterations in conformational dynamics which confer a toxic gain and/or partial loss of function. Although overexpanded ATXN2 is predominantly linked to spinocerebellar ataxia type 2 (SCA2), intermediate expansions are also implicated in amyotrophic lateral sclerosis (ALS) and parkinsonism. While the molecular intricacies await full elucidation, SCA2 presents ATXN2-associated pathological features, encompassing autophagy impairment, RNA-mediated toxicity, heightened oxidative stress, and disruption of calcium homeostasis. Presently, SCA2 remains incurable, with patients reliant on symptomatic and supportive treatments. In the pursuit of therapeutic solutions, various studies have explored avenues ranging from pharmacological drugs to advanced therapies, including cell or gene-based approaches. These endeavours aim to address the root causes or counteract distinct pathological features of SCA2. This review is intended to provide an updated compendium of ATXN2 functions, delineate the associated pathological mechanisms, and present current perspectives on the development of innovative therapeutic strategies.

## Facts

- ATXN2 is a highly abundant protein in the nervous system that is important for the maintenance of neuronal homeostasis by participating in RNA metabolism and translation, stress response, and calcium regulation.
- The *ATXN2* gene is associated with certain neurodegenerative diseases and conditions, including spinocerebellar ataxia type 2 (SCA2), amyotrophic lateral sclerosis (ALS), parkinsonism, and Machado-Joseph disease (MJD/SCA3).
- Despite the ubiquitous expression of expanded ATXN2, neurodegeneration in SCA2 occurs in a region-selective manner.
- Neuronal inclusions can be found in spared brain areas of SCA2 patients while neuronal death still occurs in their absence.
- Expanded ATXN2 is associated with SCA2 that is an incurable disease, thus patient care relies on symptomatic and supportive treatments.

## Open questions

- How is the *ATXN2* gene involved in different diseases despite the number of CAG repeats overlap among them?
- What mechanisms can explain the region-selective neurodegeneration in SCA2?
- What is the role of neuronal inclusions in these diseases? Are they harmful or cytoprotective?
- How do the various pathways and mechanisms intricately implicated in the pathogenesis of SCA2 contribute to both the onset and progression of the disease?

## Introduction

Ataxin-2 (ATXN2) is a versatile protein with key roles in several molecular, cellular, and physiological functions. For example, it binds RNA but also plays a crucial role in regulating the overall body metabolism [1–4] and the circadian rhythm [5–7]. On the other hand, the malfunctioning of ATXN2 has been associated with many diseases, especially spinocerebellar ataxia type 2 (SCA2) [8–10] and amyotrophic lateral sclerosis (ALS), but also parkinsonism, SCA1, SCA3, obesity, and diabetes [3, 11–19].

ATXN2 is a polyglutamine (polyQ) protein that typically contains a 22-glutamine tract [20]. Nevertheless, genetic instability can originate mutations that produce abnormally expanded polyQ tracts that alter the protein’s conformational dynamics, which ultimately leads to the loss of cellular homeostasis [21]. ATXN2 is widely distributed in the cytoplasm, but its expanded form tends to aggregate and shift its subcellular localization into neuronal inclusions, which is a central feature of SCA2 [22]. Whether these inclusions are cytotoxic or neuroprotective is still a matter of debate. Alterations in ATXN2 conformational dynamics also promote aberrant interactions, contributing to the acquisition of toxic functions that further contribute to neuronal dysfunction and cell death [23]. Furthermore, expanded ATXN2 can also undergo aberrant post-translational modifications (PTMs), which may be involved in disease pathogenesis [24].

**Fig. 1:**
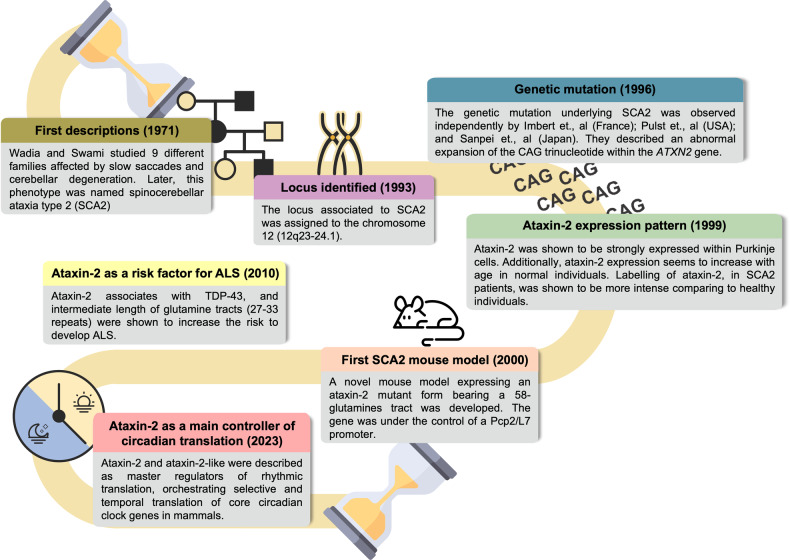
Chronology of key ataxin-2 discoveries and associated diseases. A schematic representation highlighting key milestones in the research on ataxin-2 and related diseases, including SCA2 and ALS. The timeline spans from the initial characterization of SCA2 to the latest insights into the protein’s proposed functions.

Much has been discovered about ATXN2 and its functions (Fig. 1). Therefore, this review aims to congregate and systematize the complexity of ATXN2 features by providing a detailed and comprehensive view of its molecular functions, comparing them between physiological and pathological contexts.

## Ataxin-2: distribution, subcellular localization, and structure

**Fig. 2:**
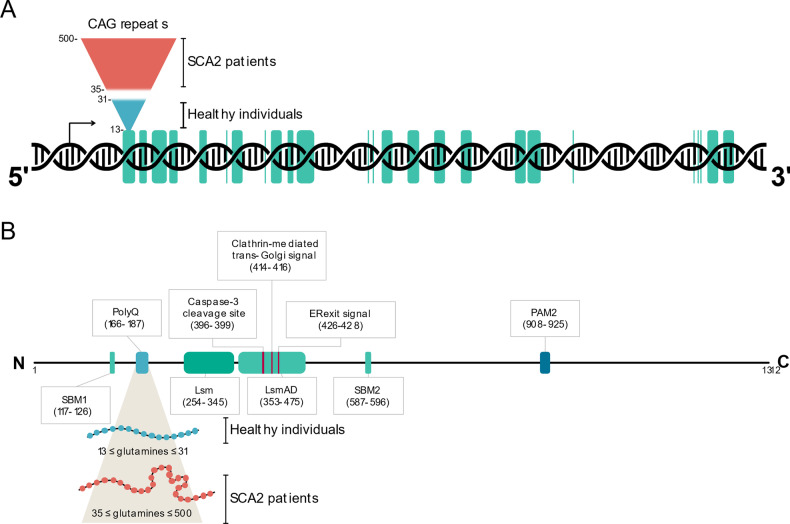
Schematic representations of the *ATXN2* gene and ATXN2 protein. The *ATXN2* gene (**A**) comprises 25 exons (green) encoding ATXN2. *ATXN2* contains a repetitive CAG region of variable length in exon 1, which may lead to the development of SCA2 when this sequence is expanded beyond 31 CAG units, with full penetrance above 35 repeats. **B** ATXN2 contains 5 protein domains beyond the polyQ tract: SBM1, Lsm, LsmAD, SBM2, and PAM2. The LsmAD domain includes a caspase-3 cleavage site, a clathrin-mediated trans-Golgi signal and an endoplasmic reticulum (ER) exit signal. Expanded *ATXN2* alleles originate ATXN2 forms with expanded polyglutamine (polyQ) sequences, which may lead to alterations in intra- and intermolecular dynamics.

The *ATXN2* gene (NCBI gene ID: 6311) encodes ataxin-2 (ATXN2) (Uniprot: Q99700), a large protein with low sequence complexity that shares no structural or functional similarities, apart from the polyQ tract, with other glutamine-rich proteins associated with neurological diseases [25]. ATXN2 is a ubiquitous protein that is expressed in every organ, reaching its highest levels of expression in the nervous system, particularly in the cerebral cortex, basal ganglia, and cerebellum, especially in Purkinje cells [8, 9, 26], where its expression increases with age [27]. ATXN2 can be found diffused within the cytoplasm, associated with polyribosomes [28], in the endoplasmic reticulum (ER) [29], Golgi apparatus [30], stress granules (SGs) [31], and, in pathological conditions, in neuronal inclusions [32]. Several isoforms of human ATXN2 have been characterized, with the canonical variant weighing 140 kDa and consisting of 1 312 amino acids. The most frequent variant in the global population contains 22 glutamines (Fig. 2). ATXN2 is a highly basic protein, except for an acidic region containing 46 amino acids (aa 254-475). It is predicted that this region defines two conserved globular domains, named Like Sm domain (Lsm; aa 254-345), containing two RNA splicing motifs (Sm1 and Sm2; aa 230-233 and 283-286, respectively), and Lsm-associated domain (LsmAD; aa 353-475). Both domains are localized in the N-terminal region of ATXN2, downstream of the polyQ stretch [24, 33, 34]. The Lsm domain enables mRNA stabilization and consequently enhances protein abundance by interacting with uridine-rich elements in the 3’-UTR of specific mRNAs [35]. The LsmAD is located downstream of the Lsm domain and harbors an ER exit signal (aa 426-428), which is required for ATXN2 exportation from the ER. Additionally, this domain contains a clathrin-mediated trans-Golgi signal (aa 414-416), required for the clathrin-mediated cleavage from the ER, Golgi apparatus, or plasma membrane [30]. The LsmAD also contains a predicted site for caspase-3 cleavage (aa 396-399) that is expected to produce a fragment with a predicted molecular weight of 41.2 kDa. This is consistent with the known production of an ATXN2 fragment with 42 kDa that was observed to be more abundant in the brain of SCA2 patients. However, the relevance of this fragment for SCA2 pathogenesis is still unknown [24, 36]. ATXN2 also comprises two proline-rich Src homology 3 (SH3) domain binding motifs, SBM1 (aa 117-126) and SBM2 (aa 587-596), which are thought to interact with proteins harboring SH3 domains, such as endophilins. Since endophilins are part of the endocytic machinery, it was suggested that ATXN2 could play a role in endocytosis [37]. The C-terminal region of ATXN2 contains a poly(A)-binding protein 1 (PABP-1) interacting motif (PAM2; aa 908-925), enabling the physical interaction between ATXN2 and PABP-1, a protein that promotes mRNA circularization during translation [38, 39]. The ATXN2/PABP-1 interaction negatively regulates translation by destabilizing the translation initiation complex, hence reducing protein abundance [28, 40]. In light of this, it was shown that ATXN2 expression downregulates mutant ataxin-3 and mitigates the disease-associated symptoms in Machado-Joseph disease/Spinocerebellar ataxia type 3 (MJD/SCA3) mice models, while the overexpression of *PABPC1* (the gene encoding PABP-1) or the ablation of the PAM2 domain in the ATXN2 protein do not produce such effects. In fact, the overexpression of *PABPC1* produces opposite results [18].

## ATXN2 post-translational modifications

There are few studies focusing on ATXN2’s PTMs, especially on reversible alterations. It was early suggested that ATXN2 undergoes ubiquitination since it colocalizes with ubiquitin in intranuclear inclusions of pontine neurons from SCA2 patients [41]. Later, it was shown that the E3 ubiquitin ligase Parkin ubiquitinates ATXN2 [42]. Another study reported that the cyclin-dependent kinase 5 (Cdk5) phosphorylates ATXN2, mainly at the N-terminal and middle regions of the protein’s sequence. This phosphorylation events seem to be important for protein turnover as they induce the proteasomal degradation of ATXN2, particularly in post-mitotic neurons, where Cdk5 is mainly active [43].

ATXN2 also undergoes proteolytic cleavage [36]. It is cleaved into two main fragments with unknown function: a 42 kDa fragment containing the polyQ region and a 70 kDa fragment that is not labelled by the polyQ-specific 1C2 antibody since it does not contain the polyQ stretch [24]. The 42 kDa fragment was found to be enriched in the brain of SCA2 patients while being practically absent in healthy individuals, a result that was also observed in 293T cells [24]. The 70 kDa fragment was detected in brain samples from both SCA2 patients and healthy individuals with no distinguishable levels. In SH-SY5Y cells, five C-terminal fragments of ATXN2 were detected, one with 70 kDa, presumably the same fragment detected in human brain samples, and four minor forms, also with unknown function [44].

The known impact of these PTMs on ATXN2’s function and SCA2 pathogenesis remains limited. In other polyQ diseases, multiple studies suggested that proteolytic cleavage of polyQ proteins could play an important role in pathogenesis [36, 45]. Aligned with this idea, the presence of an enriched 42 kDa fragment in the brain of SCA2 patients implies the potential significance of ATXN2 cleavage in pathogenic processes. However, in COS-1 cells, truncated N-terminal forms of ATXN2, mimicking the 42 kDa fragments observed in patients, did not increase cytotoxicity. Conversely, expanded full-length ATXN2 induced cell death. Furthermore, these truncated fragments did not form intracellular inclusions and remained diffusely distributed in the cytoplasm, highlighting the importance of the ATXN2 C-terminus in initiating aggregation in SCA2 [46].

## ATXN2 interacting partners and associated functions

**Fig. 3:**
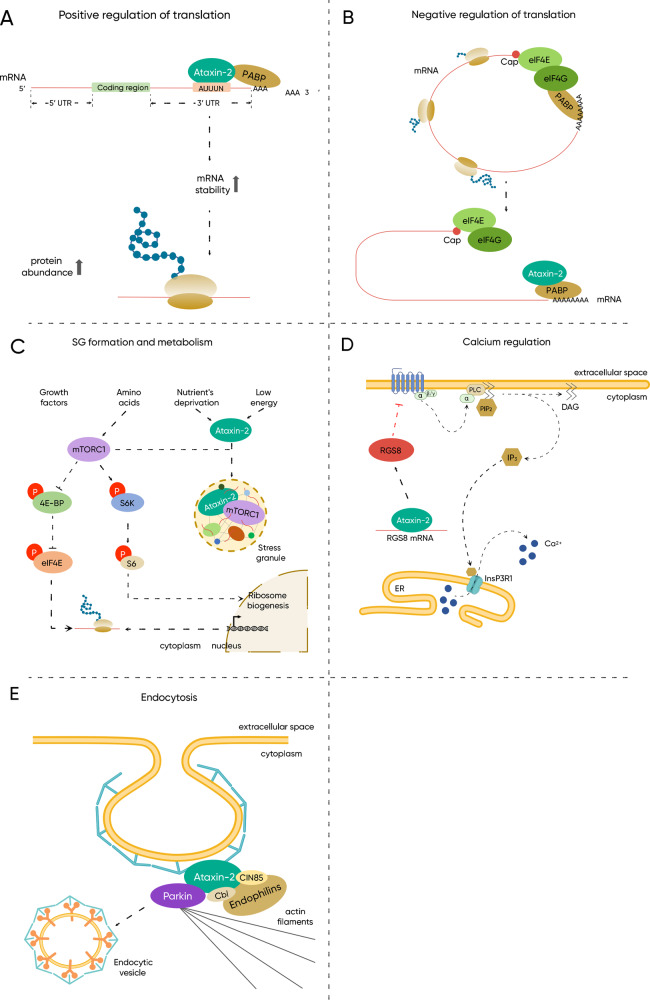
Molecular functions of ATXN2. ATXN2 is implicated in many distinct cellular processes, including (**A**) the positive regulation of mRNA translation by directly binding to and stabilizing mRNAs and, conversely, (**B**) the negative regulation of mRNA translation by binding to PABP-1 and impairing the formation of the translation initiation complex. **C** ATXN2 is a regulator of metabolism as it can sequester mTORC1 into SGs under nutrient deprivation conditions, thus hampering downstream signalling and, consequently, protein synthesis and cell growth. **D** ATXN2 was implicated in calcium homeostasis maintenance by negatively regulating mGluR1 through stabilization of the mRNA of its antagonist RGS8. **E** ATXN2 is also associated with endocytosis by interacting with endophilins, Cbl, and CIN85, and was shown to influence the internalization rate of EGFR.

The ATXN2 protein has been experimentally shown to interact with at least 296 components in human samples (BioGRID ID: 112218). The determination of ATXN2 functions (Fig. 3), which are not fully understood, has been approached through three distinct methods: (i) by observing the cellular and molecular consequences of modulating ATXN2 expression, either through overexpression or silencing of the *ATXN2* gene, (ii) by using bioinformatic tools to analyse structural similarities between ATXN2 and other known proteins that share previously characterized motifs and domains, and (iii) by characterizing the intermolecular interactions involving ATXN2.

### RNA metabolism and translation

ATXN2 is an RBP involved in multiple steps of RNA metabolism. Besides the direct interaction with RNA, ATXN2 also associates with partner proteins actively involved in RNA metabolism. For example, ATXN2 interacts with proteins associated with alternative splicing, including A2BP1, RNA-binding motif protein 9 (RBM9), and the RNA-binding protein with multiple splicing (RBPMS). Despite RNA splicing predominantly occurring in the nucleus and ATXN2 being primarily located in the cytoplasm, the interaction between ATXN2 and A2BP1, RBM9, and RBPMS hints at a potential involvement in modulating RNA splicing. This potential role may involve sequestering these proteins in the cytoplasm, thereby limiting their availability within the nucleus [47, 48]. However, the presumed role of ATXN2 in RNA splicing demands more in-depth characterization, given the distinct cellular compartments in which ATXN2 expression and RNA splicing occur [34].

ATXN2 is also suggested to play important roles in the regulation of translation. The Lsm, LsmAD, and PAM2 domains have been demonstrated to independently interact with polyribosomes located in the rough ER [29]. Moreover, the ATXN2/PABP-1 interaction negatively regulates mRNA translation [18, 28]. On the other hand, as previously mentioned, ATXN2 can enhance the stability of mRNA molecules and increase protein abundance by binding to uridine-rich elements within the 3’ UTRs through an Lsm-mediated interaction [35].

ATXN2 may also participate in the regulation of mRNA poly(A) tail length by inhibiting the activity of the poly(A) nuclease (PAN) deadenylation complex. This inference is drawn from findings indicating that Pbp1, the yeast homolog of ATXN2, collaborates with Pab1, the yeast homolog of PABP-1, to hinder the PAN deadenylation complex’s access to the mRNA’s poly(A) tail. [49, 50]. ATXN2-mediated inhibition of mRNA decay induced by deadenylation could thus promote translation since longer poly(A) tails facilitate RNA looping [51].

Finally, it was suggested that Drosophila’s Atx2 could be implicated in long-term olfactory habituation by functioning with Fragile X messenger ribonucleoprotein 1 homolog (dFMR1) and miRNA pathway proteins (Ago1 and Me31B – Drosophila’s DDX6 homolog). This inference is supported by the fact that Atx2 knockdown enhances the expression of a translational reporter carrying the 3’UTR of Ca^2+^/calmodulin-dependent protein kinase II (CaMKII), in olfactory neurons. These studies identified Atx2 as a component of the miRNA pathway [52, 53].

### Stress granules formation

Stress granules (SGs) are transient membraneless foci that dynamically assemble and disassemble upon the presence and resolution of stress stimuli, respectively [54]. Their composition includes hundreds of proteins, and their components may differ depending on the stressor [55]. Many components are commonly observed in SGs, including poly(A)-mRNAs, 40S ribosomal subunits, PABP-1, eIF4A, eIF4B, eIF4E and eIF4G [56, 57]. Additionally, SGs are rich in RBPs that directly impact mRNA metabolism, such as Ras-Gap SH3-binding protein 1 (G3BP1), survival motor neuron protein (SMN), T-cell restricted intracellular antigen 1 (TIA1), TIA-1-related protein (TIAR) and Staufen-1 [58–60].

ATXN2 is a known component of SGs, but its role within these structures is contradictory [31, 35, 39]. A study using *ATXN2*-knockout cells reported that ATXN2 was not essential for SGs assembly upon a stress stimulus with sodium arsenite. Nevertheless, ATXN2-like (ATXN2L), a protein that shares great similarity with ATXN2 and which is also a component of SGs, could have a redundant role in SGs dynamics and compensate for the absence of ATXN2 [61]. However, an opposing study showed that *ATXN2* downregulation impairs SGs assembly, raising the question of whether ATXN2L can really compensate for the absence of ATXN2 [31]. Thus, more studies are needed to clarify the complete role of ATXN2 in SGs dynamics.

### Endocytosis and cytoskeleton reorganization

ATXN2 has been proposed to participate in endocytosis and cytoskeleton reorganization by interacting with key proteins from the endocytosis machinery, including endophilins A1 and A3, the E3 ubiquitin ligase Cbl, the adaptor Cbl-interacting protein of 85 kDa (CIN85), and the protein kinase Src. Endophilins A1 and A3 are integral components of a protein complex that facilitates the curvature formation of the plasma membrane, hence allowing cargo internalization. As an example, upon activation of the epidermal growth factor receptor (EGFR), endophilins A combine with Cbl, CIN85, and Src to facilitate EGFR internalization. ATXN2 has been demonstrated to delay the internalization of EGFR, while *ATXN2*-knockout cells exhibit an accelerated rate of receptor internalization. This implies that ATXN2 may function as a regulator of endocytosis [37].

Endocytosis is initiated by the activation of a protein complex regulated by ubiquitination and intricately linked to actin filaments. The orchestration of ubiquitin-mediated activation of endocytosis is believed to be facilitated by Parkin, as it targets key components such as endophilins, along with its primary binding partners dynamin and synaptojanin-1. These components play crucial roles in the complex process of endocytosis [62]. Moreover, Parkin extends its ubiquitinating activity to target the epidermal growth factor receptor substrate 15 (Eps15), another protein involved in endocytosis [63]. The pronounced functional involvement of Parkin in endocytosis, coupled with the documented interaction between ATXN2 and Parkin [42], supports the idea of ATXN2 playing a significant role in this process.

### Calcium-mediated signaling and homeostasis

ATXN2 has been implicated in the regulation of Ca^2+^-mediated signaling through its interaction with the regulator of G protein signaling 8 (RGS8) mRNA. RGS8 acts by stimulating the GTPase activity of the GPCR alpha subunit, turning it into its GDP-bound inactive form, and, thus, blocking signal transduction. Consequently, Ca^2+^ release into the cytosol is interrupted [64]. It is thought that ATXN2 regulates *RGS8* mRNA levels in Purkinje cells by stabilizing it. This in turn negatively regulates the metabotropic glutamate receptor subtype 1 (mGluR1)-mediated signaling, preventing potential consequences of excitotoxicity caused by exacerbated intracellular levels of Ca^2+^ []. Accordingly, it was found that polyQ-expanded ATXN2 loses the ability to interact with *RGS8* mRNA and that *RGS8* expression levels are decreased in the cerebellum of SCA2 mice and lymphoblasts of SCA2 patients. This evidence suggests that ATXN2 is important to stabilize *RGS8* and protect it from degradation, but the alterations in conformational dynamics due to the overexpanded polyQ tract lead to a loss of function of ATXN2, which can no longer stabilize *RGS8* mRNA and it ends up being degraded [65]. Another study also suggests that ATXN2 is involved in Ca^2+^-mediated signaling by acting as a regulator of factors involved in Ca^2+^-dependent pathways. In this study, *Atxn2*-KO mice presented altered levels of Ca^2+^ signaling components, especially those of PLCβ (*Plcb3*, *Plcb4*), IP_3_ metabolism enzymes (*Inpp5a*, *Itpka*), and Ca^2+^/Calmodulin-dependent kinases (CaMK) (*Camk2a*, *Camk2g*, *Camk4*), as well as CaMK-kinases (CaMKKs) (*Camkk1*) [66]. Therefore, ATXN2 could be important to regulate the abundance of transcripts involved in Ca^2+^-dependent pathways, being itself important to indirectly regulate Ca^2+^ signaling.

### Cellular metabolism

The mammalian target of rapamycin (mTOR) signaling pathway controls protein synthesis by stimulating the initiation of mRNA translation, but since this is an energy-demanding anabolic process, it only occurs when nutrients are abundant and in the absence of bioenergetic deficits [67]. Nutrient deprivation and cellular bioenergetic deficits elicit the transcriptional activation of *ATXN2*, increasing its abundance. ATXN2 then inhibits mTORC1 signaling by sequestering mTORC1 into SGs, hampering downstream signaling, consequently inhibiting the initiation of translation and ribosomal biogenesis [61, 68]. Altogether, ATXN2 seems to contribute to the slowing of the cell’s anabolic state under starvation-induced stress.

The role of ATXN2 in cellular metabolism seems also to be aligned with the hypothesis that ATXN2 regulates food intake and body weight by acting as a nutritional and energetic sensor in a very complex network [2]. In this context, it was found that *Atxn2*-KO mice display hepatic steatosis and abdominal obesity, accompanied by insulin resistance in the liver and cerebellum [2–4]. Recently, it was shown that overexpressing *ATXN2* specifically in the hypothalamus prevented high-fat diet-induced obesity and insulin resistance. Moreover, ATXN2 re-establishment in *Atxn2*-KO mice improved metabolic dysfunction without changing body weight [1].

### Circadian rhythm

ATXN2 was also implicated in the regulation of circadian rhythm (Fig. 1). The molecular basis of the circadian rhythm consists in the oscillating expression of the circadian clock component genes [69]. The Drosophila Atx2 (the fly ATXN2 homolog) is part of the circadian clock complex by associating with Tyf and acting as a post-transcriptional co-activator for the translation of *per* mRNA. Tyf is recruited to the Lsm/LsmAD domain of Atx2 by the adaptor protein Lsm12, and this association facilitates the physical interaction between Tyf, the 5’ cap-binding translation initiation factors, and PABP-1. Consequently, the translation of *per* mRNA is activated in the brain pacemaker neurons, and Per maintains a 24-hour periodicity circadian behavior [5–7]. The activation of *per* translation mediated by Atx2 is a PABP-1-dependent mechanism, as the deletion of the PAM2 domain in the Drosophila Atx2 decreased the abundance of the Per protein and consequently suppressed the fly’s behavioral rhythms [6].

In mammals, ATXN2 has also been studied in the context of the circadian rhythm, albeit to a lower extent due to its enhanced complexity. In jet lag experiments, *Atxn2*-KO mice exhibited a delayed adaptation period to a new light/dark cycle, indicating impairment in the clock mechanism, despite the absence of alterations in PER1 and PER2 immunoreactivity [70]. However, the impact of the Atxn2 deletion on the circadian rhythm in mice appears to be more subtle than that observed in Drosophila. This subtlety might be explained by the presence of the orthologue Atxn2l, found in all vertebrates with the exception of birds, which could be compensating for the absence of Atxn2, at least to some extent.

## Spinocerebellar ataxia type 2

**Fig. 4:**
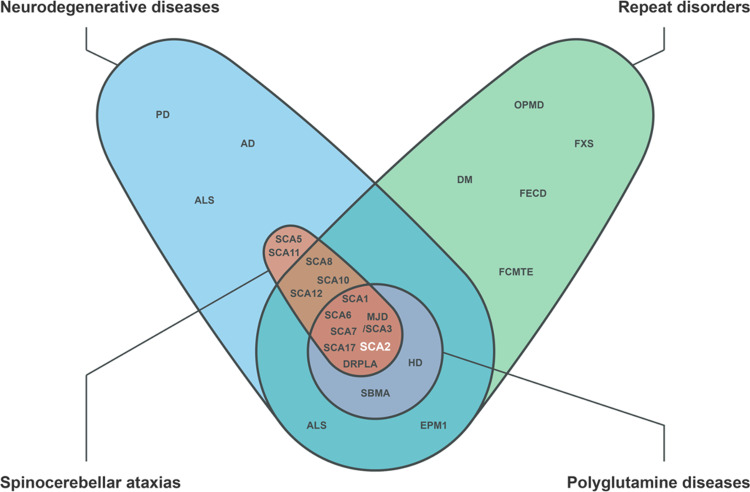
Polyglutamine diseases and spinocerebellar ataxias at the intersection of neurodegeneration and repeat expansion mutations. Polyglutamine diseases are a group of neurodegenerative diseases, a wider collection of neurologic disorders that includes Alzheimer’s disease (AD), Parkinson’s disease (PD), amyotrophic lateral sclerosis (ALS), among many others. PolyQ diseases are hereditary diseases caused by expansion of CAG microsatellites in the coding region of the respective causative genes. The group comprises Huntington’s disease (HD), spinal and bulbar muscular atrophy (SBMA) and seven types of spinocerebellar ataxias (SCA): SCA1, 2, 3 (also known as Machado-Joseph disease, MJD), 6, 7, 17 and dentatorubral-pallidoluysian atrophy (DRPLA). SCAs are autosomal dominant neurodegenerative disorders typically manifested by progressive ataxia, and not all are associated with polyQ expansions. Among the ones for which a causative mutation has been described, some are caused by nucleotide repeat expansion outside coding regions (for example SCA8, 10 and 12) and others by conventional mutations (examples include SCA5 and SCA11). While neurodegenerative disorders other than polyQ diseases and SCAs are also caused by repeat expansion mutations (including progressive myoclonic epilepsy type q, EPM1), not all repeat expansion diseases involve a clear neurodegenerative profile. Examples include myotonic dystrophy (DM), familial cortical myoclonic tremor with epilepsy type 1 (FCMTE), Fuchs endothelial corneal dystrophy (FECD), fragile X syndrome (FXS) and oculopharyngeal muscular dystrophy (OPMD). Only a portion of ALS cases are known to be associated with repeat expansions.

Spinocerebellar ataxia type 2 (SCA2) is a rare autosomal neurodegenerative disease belonging to the group of polyQ diseases, which are caused by an abnormal expansion of the trinucleotide CAG in their respective causative genes [36]. SCAs are heterogenous genetic ataxias with an autosomal dominant inheritance pattern, and although a total of more than 50 types have been described, only six (SCA1, -2, -3, -6, -7, and -17) and DRLPA, also classified as a SCA, are caused by a CAG repeat expansion in the coding region of their associated genes (Fig. 4) [71, 72].

SCA2 was first documented in India by Wadia and Swami, in 1971, who examined 16 patients from nine families and reported early and marked slow saccades (simultaneous movement of both eyes towards a fixed point) associated with cerebellar degeneration [73] (Fig. 1). Later, a high prevalence of similar symptoms was described in Spanish descendants in Holguín, Cuba [74].

In 1996, the causative mutation of SCA2 was identified independently by three laboratories in France, the USA, and Japan as an abnormal expansion of the CAG triplet repeat region present in the first exon of the *ATXN2* gene (Fig. 1). Wild-type alleles contain between 13 and 31 CAG repeats, with more than 90% of the population carrying a 22 CAG tract [20]. The frequency of this allele can be attributed to the genetic stability of this region that is conferred by the insertion of one or two CAA codons between the CAG repeats, following the pattern (CAG)_8_-CAA-(CAG)_4_-CAA-(CAG)_8_. Conversely, SCA2 alleles usually present an uninterrupted and pure CAG tract that is expanded beyond 32 CAG repeats, with full disease penetrance above 35 CAGs [75]. The absence of CAA interruptions in SCA2 alleles is thought to contribute to genetic instability, increasing their propensity to expand in each generation [21]. This is commonly associated with earlier onset and increased severity of the disease. This phenomenon, termed anticipation, enables the emergence of pediatric cases of SCA2 associated with very large CAG expansions, up to 230-500 CAG units [76].

### Neuropathological features and clinical presentation

*Post-mortem* studies performed in SCA2 patients revealed a significant atrophy of the cerebellum, brainstem, frontal lobe, and nearly all cranial nerves. A reduction in white matter was also described in the cerebrum and cerebellum, along with depigmentation of the midbrain *substantia nigra* [15, 16, 77, 78]. Histopathological studies demonstrated a pronounced loss of Purkinje cells, although several studies have shown that neuronal loss is widespread[77]. In line with this data, magnetic resonance imaging (MRI) scans revealed severe cerebellar volume loss in both white and gray matter, with atrophy of the pons, medulla oblongata, spinal cord, parietal cortex, and thalamus [79, 80].

These neuropathological observations translate into the main clinical features of SCA2 patients, of which gait ataxia is the most noticeable symptom. The lack of motor coordination results from the progressive degeneration of the cerebellum and its associated circuits [81, 82]. Cerebellar degeneration leads to other symptoms that include appendicular ataxia with instability of stance, dysarthria, and oculomotor deficits presented by nystagmus and ocular dysmetria [83, 84]. Variant phenotypes of motor incoordination have been observed, including L-DOPA-responsive parkinsonism, which may be explained by the *substantia nigra* degeneration, and motor features of ALS [15, 16, 85, 86]. Other manifestations result from the neurodegeneration of the pons, basal ganglia, cerebral cortex, and other regions of the nervous system. For instance, slow or absent saccadic movements are a predominant ocular feature of SCA2, originating primarily from the degeneration of neurons in the oculomotor brainstem [87]. Frequently, ataxia onset coincides with painful muscle cramping, presumably resulting from a hyper-excitability state of the motor neurons caused by collateral sprouting processes in their distal portions after subtle axonal damage [88, 89]. Other symptoms include dystonia, myoclonus, neuropathy, muscle spasticity, and cognitive, emotional, and behavioral problems caused by neurodegeneration of the frontal lobe [89, 90].

## The abnormal *ATXN2* expansion

**Fig. 5:**
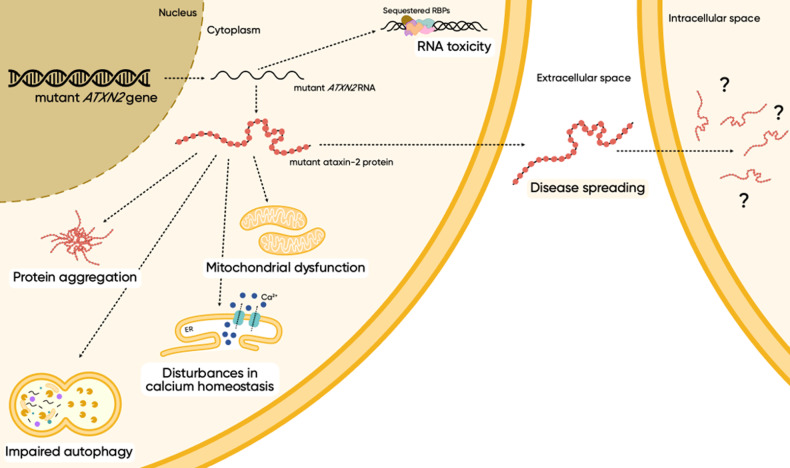
Molecular mechanisms proposed to be involved in SCA2 pathogenesis. Both repeat-expanded sense and anti-sense transcripts of *ATXN2* can form hairpin structures and cause toxicity, presumably by sequestering RBPs into RNA foci. The translation of the sense transcript results in a polyQ-expanded ATXN2 protein that is prone to adopt a β-sheet-rich structure and form cytoplasmic insoluble aggregates that eventually recruit other proteins, such as ataxin-1, ataxin-3, and TBP. The accumulation of SQSTM1 and LC3-II indicates a dysfunction of the autophagic pathway, suggesting that neurons struggle to clear out damaged and aggregated proteins, leading to an overall loss of proteostasis. Additionally, low levels of PINK1 in SCA2 patients indicate mitochondrial dysfunction and enhanced oxidative stress, which can contribute to neuroinflammation and neuronal death. Mutant ATXN2 aberrantly interacts with the ER InsP_3_R1 and increases its sensitivity to IP_3_, resulting in high calcium leakage into the cytosol that can trigger excitotoxicity and neurodegeneration. Interneuronal transfer of mutant ATXN2 could constitute a mechanism contributing to SCA2 progression, whereby cells that are chiefly affected disseminate toxic species to the vicinity, a process known as “disease spreading”.

The current perspective is that ATXN2 undergoes a toxic gain and partial loss of function, affecting many systems and pathways thought to be involved in the molecular events of neurodegeneration (Fig. 5) [32]. The proposed mechanisms of pathophysiology include protein aggregation, aberrant interactions, autophagy impairment, RNA-mediated toxicity, enhanced oxidative stress, cell signaling alterations, and disturbances in calcium homeostasis. Recently, aging was also implicated in SCA2 pathogenesis as mutant ATXN2 seems to be more prone to aggregate in aged animals, which also display a more pronounced loss of neuronal markers [91].

### Protein aggregation

Abnormally expanded ATXN2 shifts its conformation to a β-sheet-rich structure, rendering it more prone to form insoluble aggregates with amyloid fibrillar morphology that accumulate in neurons as inclusion bodies [22]. In SCA2 patients’ brains, aggregates are mainly found in the cytoplasm, although intranuclear aggregates have also been reported [92]. The aggregates were shown to contain other proteins, including ataxin-1, ataxin-3, and the TATA box-binding protein (TBP) [93], but, contrasting with the aggregates found in other polyQ diseases, in SCA2 they are rarely ubiquitinated [41, 94].

The toxic nature of aggregates and their contribution to neuronal death are still topics of controversy. While some studies report a positive correlation between the presence of polyQ proteins aggregates and neurodegeneration [95, 96], others suggest that intracellular aggregates may be cytoprotective [94, 97]. As for SCA2, studies in cellular models found that the expression of polyQ-expanded ATXN2 led to increased cell death compared to wild-type ATXN2. However, aggregate formation was not needed for cell death to occur [30]

### Autophagy impairment

Autophagy is a selective lysosome-mediated degradation process that was shown to be important in the clearance of aggregates containing polyQ proteins [98]. In cells, the degradation of misfolded proteins can also occur through the ubiquitin-proteasome system (UPS); however, proteins containing long polyQ tracts are not efficiently degraded by eukaryotic proteasomes [99]. Therefore, autophagy is crucial in the context of polyQ diseases, to degrade both soluble and aggregated forms of expanded proteins [100]. However, autophagy impairment was described as a feature of polyQ diseases [101]. In SCA2, it was found that patients’ fibroblasts show an accumulation of the autophagic markers sequestosome-1 (p62/SQSTM1) and light chain 3 isoform II (LC3-II), indicating a reduction in the autophagic flux [102]. Recently, a study reported altered levels of SQSTM1 and LC3B-II upon expression of mutant *ATXN2* in N2a cells. Moreover, an abnormal accumulation of these markers was detected in SCA2 patients’ striatum and cerebellum [54].

### RNA-mediated toxicity

Expanded repeat-containing RNAs are thought to induce toxicity by aberrantly interacting with RBPs. It is hypothesized that transcripts harboring expanded CAG/CUG repeats can undergo 3D conformational changes and form hairpin structures that can sequester RBPs and prevent them from performing their normal functions [103–105].

Recently, it was discovered that an aberrant natural antisense transcript (NAT)-based process may be involved in SCA2 pathogenesis. NATs are antisense RNAs transcribed from the opposite, non-template, DNA strand, which can regulate gene expression at multiple levels, while their dysregulation has been associated with disease development [106]. The *ATXN2* locus was shown to be transcribed bidirectionally, originating the mRNA encoding ATXN2 and an antisense transcript named *ATXN2-AS*. The SCA2-associated *ATXN2-AS* transcript contains an abnormally expanded CUG sequence and was found to be present in the brain of SCA patients, as well as in patient-derived fibroblasts, induced pluripotent stem cells, and neural stem cells [107]. The same study reported that the abnormally expanded *ATXN2-AS* transcript was toxic in a cellular model of SCA2 and formed RNA foci in the cerebellar Purkinje cells of SCA2 transgenic mice and in SCA2 patients’ brains.

Later, it was shown that the expanded *ATXN2* sense transcript could also induce neurotoxicity and form RNA foci in cellular and mouse models of SCA2, which were also detected in the brain of an SCA2 patient. It was suggested that the expanded *ATXN2* transcript aberrantly interacts with the transducin β-like protein 3 (TBL3), an RBP that is required for rRNA processing. The consequent failure of rRNA maturation could prevent the assembly of ribosomal proteins into ribosomal ribonucleoprotein subunits, hence contributing to neuronal death [108].

Taking all observations together, RNA-mediated toxicity, in combination with putatively toxic and soluble ATXN2 oligomers resulting from ATXN2 self-assembly, may explain the neurodegeneration observed in those cases where protein aggregation does not correlate with cell death [94, 97, 109, 110].

### Enhanced oxidative stress

Oxidative stress arises as a result of an imbalance between the production of ROS and the biological system’s ability to detoxify the reactive intermediates, which is implicated in several neurodegenerative diseases [111]. Global transcriptome analyses suggest that ATXN2 may positively regulate PTEN-induced kinase 1 (PINK1) by interfering with its mRNA processing, as it was shown that *Atxn2*-KO mice present reduced expression of PINK1, whereas blood samples from SCA2 patients show increased levels [112]. PINK1 is a mitochondrially targeted serine/threonine kinase thought to play a neuroprotective role by preventing mitochondrial dysfunction-mediated damage, oxidative stress, and apoptosis [113, 114]. The increased levels of PINK1 in SCA2 may indicate disturbances in mitochondrial homeostasis and an impaired response to oxidative stress. In line with this, fibroblasts from SCA2 patients were found to abnormally express two antioxidant enzymes: (i) mRNA and protein levels of the Cu/Zn superoxide dismutase (SOD1) enzyme were found to be increased, while (ii) the catalase enzyme was significantly downregulated [115]. This could lead to an abnormal accumulation of H_2_O_2_ due to its impaired conversion into non-toxic molecules, contributing to stress increase. However, the mechanisms through which mutant ATXN2 exacerbates oxidative stress were not addressed in these studies. Nonetheless, another study shed some light on the pathways driving oxidative stress in SCA2 showing that mutant ATXN2 associates with gp91, a catalytic subunit of the NADPH oxidase that produces a superoxide free radical, which produced an oxidative wave that induced mitochondrial distress and DNA damage [116].

### Cell signalling alterations

It was hypothesized that disturbances in cell signaling mechanisms may underlie pathological processes and contribute to neurodegeneration [32]. In this line, alterations in the activity of signaling proteins have been described in several SCAs [117, 118], suggesting that signaling disturbances could also be a feature of SCA2. In fact, an SCA2 mouse model displayed severely reduced levels of metastasis suppressor protein 1 (MTSS1), a suppressor of the Src family of non-receptor tyrosine kinases (SFK). In this study, the polyQ-expanded ATXN2 was suggested to block MTSS1 translation and, consequently, increase SFK signaling, leading to a reduction of Purkinje cells’ arborizations and basal firing rates [31].

### Disturbance of calcium homeostasis

Calcium ions serve as second messengers and play a fundamental role in cell signaling. Additionally, specific regulatory proteins and enzymes rely on Ca^2+^ to carry out their functions. Neurons rely on a tight control of intracellular Ca^2+^ levels, and high levels of Ca^2+^ within the cytosol can disrupt neuronal signaling and cellular homeostasis, potentially leading to synaptic loss and neuronal death [32].

The ATXN2-binding protein 1 (A2BP1), also known as RNA-binding protein fox-1 homolog 1 (RBFOX1; Uniprot: Q9NWB1), is an RBP that interacts with the C-terminus of ATXN2 [119]. A2BP1 is involved in the alternative splicing of the NMDA receptor GluN1 subunits [120]. Under certain circumstances, the splicing regulator A2BP1 can target exon 5 of GluN1 [120], suggesting that A2BP1 may induce neurons to enter a state of excitotoxicity. In SCA2, the expanded ATXN2 may lose the ability to interact with A2BP1, promoting excitotoxicity and potentially explaining the cell type-specific neuronal death observed in SCA2 patients [119]. It has also been shown that SCA2-iPSC-derived neurons exhibit an altered gene expression profile compared to healthy iPSC-derived neurons, particularly in glutamate receptor signaling. SCA2-iPSC-derived neurons show alterations in the expression of glutamate receptor-related genes, such as *GRIA4* and *GRM3*. These changes contribute to significantly increased levels of intracellular Ca^2+^ upon glutamate treatment [121].

Transcriptome analyses revealed a decrease in both mRNA and protein levels of RGS8, a protein implicated in mGluR1 signaling, in the cerebellum of an SCA2 animal model and in lymphoblastoid B cells from SCA2 patients [65]. The absence of negative regulation of mGluR1 signaling may lead to an increased concentration of Ca^2+^ in the cytosol, promoting excitotoxicity and neuronal death. Moreover, expanded ATXN2, but not its wild-type form, interacts with the type 1 inositol 1,4,5-trisphosphate receptor (InsP_3_R1) in the ER membrane. This aberrant interaction leads to increased sensitivity to IP_3_, hyperactivation of InsP_3_R1, and, subsequently, the release of high amounts of Ca^2+^ from the ER into the cytosol [23]. The overloaded Ca^2+^ ions may be pumped into the mitochondria, leading to mitochondrial swelling and further rupture of their outer membranes. Ultimately, mitochondrial rupture could release cytochrome *c* into the cytosol, triggering apoptosis and neuronal death [23, 32].

Another study found changes in the expression of several Ca^2+^ channels and transporters (*Itpr1*, *Ryr3*, *Atp2a2*, *Atp2a3*, and *Trpc3*) in the *Atxn2*-CAG100-Knock-In mouse model. Additionally, the expression of genes related to IP_3_ metabolism (*Plcg1*, *Inpp5a*, *Itpka*) and Ca^2+^/Calmodulin-dependent kinases (*Camk2a*, *Camk4*) was found to be altered as early as 3 months of age. Interestingly, *Atxn2*-CAG100-Knock-In mice exhibited specific downregulation of *Gria3* (GluA3), *Grm1* (mGluR1), and *Grm4* (mGluR4), which was not observed in *Atxn2*-KO mice. This suggests that ionotropic and metabotropic glutamate receptors could be affected by Atxn2 gain-of-function. *Atxn2*-CAG100-Knock-In mice showed increased Sam68 protein levels, suggesting that *Nrxn1* pre-mRNA splicing could be directed towards selective isoforms. Because Neurexin-1 is an important protein involved in the maintenance of synaptic structure, aberrant splicing of *Nrxn1* could indicate defective connectivity between granule and Purkinje neurons [66].

### Disease spreading

As suggested in the pathogenesis of Alzheimer’s disease (AD) and Parkinson’s disease (PD), it is reasonable to hypothesize that the progression of SCA2 may traverse the brain in a stepwise manner from one anatomical region to the next [122–124]. Neurodegeneration in SCA2 has been proposed to occur in anatomically interconnected brain areas, following the neuronal fibers outlined by tracing studies in non-human primates [122–125].

Possible explanations for the neuron-to-neuron propagation of neurodegenerative alterations may involve mechanisms such as transsynaptic and interneuronal spread of aggregates of disease-associated proteins, potentially through tunneling nanotubes or the prion-like propagation of these proteins [124, 126]. Examining the spatiotemporal expansion of neurodegenerative processes in SCA2 could enable an accurate reconstruction of the involved mechanisms and identify the origin of SCA2 pathology. Crucially, these regions may serve as potential targets for therapeutic interventions that could impede or delay the spread of SCA2 by disrupting the stepwise cascade of neurodegeneration [127].

### Alterations in SGs dynamics

Beyond ATXN2, numerous other proteins implicated in the etiology of neurodegenerative diseases are identified as constituents of SGs. This includes, for example, the survival motor neuron 1 protein (linked to muscular atrophy), the fragile X mental retardation protein (associated with fragile X syndrome), Tau (associated with AD), as well as FUS, angiogenin, optineurin, and TDP-43 (all associated with ALS). This observation suggests a potential connection between SGs and the onset of neurodegenerative diseases [128]. ATXN2 physically interacts with TDP-43 and, in the context of SCA2, expanded ATXN2 is responsible for the mislocalization of TDP-43. Being both ATXN2 and TDP-43 components of SGs, it is reasonable to hypothesize that SGs dynamics could be affected in SCA2 [14, 128]. ATXN2 was also shown to interact with Staufen-1 [102], a component of SGs, and important for their formation and disassembly [60]. In SCA2 patient cells and animal models, an overabundance of Staufen-1 was observed [129], which might impair SGs formation upon stress [60]. On the other hand, Staufen-1 downregulation, which facilitates SGs formation upon stress, was shown to mitigate SCA2-associated pathology [102].

## ATXN2 in other diseases

Studies have suggested that expanded *ATXN2* increases the risk of developing other diseases, such as ALS and PD (Fig. 1). One of ALS’s characteristics is the presence of ubiquitinated cytoplasmic inclusions of TDP-43 in patients’ neurons [130]. Interestingly, it has been observed that TDP-43 mislocalizes in SCA2 and ATXN2 mislocalizes in ALS [14, 131]. In ALS, some patients display a CAG tract of intermediate length, consisting of approximately 27–33 CAG repeats (below the SCA2 repeat threshold) in the *ATXN2* gene [86]. Moreover, ATXN2 is a potent modifier of TDP-43 toxicity in animal and cellular models [14]. *ATXN2* downregulation in a mouse model of ALS increases the lifespan and motor performance while reducing the accumulation of TDP-43 into inclusions [132]. One of the proposed mechanisms that could explain TDP-43 toxicity is that, upon stress, intermediate-length ATXN2 exacerbates the activation of multiple caspases, including caspase-3. This produces an increased amount of TDP-43 C-terminal fragments, which induce toxicity [133].

Another study showed that a longer CAG expansion in the *ATXN2* gene (32-39 repeats) was found in some patients with sporadic ALS [86]. However, this overlap of CAG expansions between ALS and SCA2 does not explain the differences between clinical features of both diseases, suggesting that other factors could influence the emergence of either disease [134]. A special case in one family was described, in which a paternal uncle was diagnosed with ALS and displayed a tract of 39 CAGs in the *ATXN2* gene, whereas his niece was diagnosed with SCA2 and had a tract of 40 CAGs. The uncle did not display major cerebellar symptoms or cerebellar atrophy [131].

Another link between ALS and intermediate-length *ATXN2* has been established via the fused in sarcoma (*FUS*) gene, which causes ALS when mutated. ATXN2 and FUS co-localize within the ER and Golgi compartments. It was observed that mutant FUS induces ER stress and Golgi apparatus fragmentation, which are exacerbated by intermediate-length ATXN2. Moreover, it seems that ATXN2 preferentially interacts with mutant FUS rather than wild-type FUS, increasing its stability and contributing to FUS aggregation [132, 135].

ATXN2 has also been linked as a risk factor for developing frontotemporal-dementia-ALS (FTD-ALS), when the *C9orf72* gene is mutated. The *C9orf72* gene is responsible for most cases of familial forms of ALS. It seems that the (GGGGCC)_n_ hexanucleotide repeat expansion in the *C9orf72* gene, plus the intermediate-length CAG expansion in the *ATXN2* gene, potentiate the development of an ALS variant characterized by frontotemporal-dementia [136–138].

In PD, it was described that some patients that harbour 36-37 CAG repeats in the *ATXN2* gene (above the threshold to develop SCA2) may not present pronounced cerebellar symptoms but instead parkinsonian features that are responsive to L-DOPA. The difference between these PD and SCA2 patients seems to be the interruption of the CAG expansion with redundant CAA units in PD, whereas SCA2 patients usually contain a pure CAG tract in the *ATXN2* gene [139–141].

Parkin mutations are related to most cases of familial forms of early-onset PD [142]. Parkin interacts with the N-terminal part of ATXN2, and it ubiquitinates both normal and expanded ATXN2. The overexpression of parkin mitigates the toxicity caused by expanded ATXN2. Additionally, in SCA2 patients, Purkinje neurons are less immunoreactive to parkin. It is possible that parkin is being sequestered to mutant ATXN2 aggregates, preventing it from performing its normal functions and impacting SCA2 pathogenesis [42].

In MJD/SCA3, an association was described for intermediate-length polyQ ATXN2 (27–32 CAG repeats) and an earlier age of onset [19]. Additionally, a single nucleotide polymorphism (rs7969300) that modulates the age of onset of MJD/SCA3 has been identified in *ATXN2* [13]. At a molecular level, it was shown that ATXN2 levels are reduced in animal models of the disease and in *post-mortem* brain tissue from MJD/SCA3 patients. Accordingly, the reestablishment of ATXN2 levels reverted several pathological hallmarks in different mouse models of MJD/SCA3 [18]. The underlying mechanism could be linked to the interaction between ATXN2 and PABP-1, which may regulate the translation of ATXN3, the causative protein of MJD/SCA3 [18, 28].

ATXN2 dysfunction has also been linked to obesity and diabetes [1, 2]. The *Atxn2*-KO mouse model gained more weight as compared to wild-type animals; however, these differences were only observed in a high-fat food regime [17]. In this mouse model, it was found that there was a downregulation of the insulin receptor in the cerebellum and liver, which was linked to abdominal obesity and hepatosteatosis [3]. Polyphagia and obesity have been reported in an SCA2 family in the middle stage of the disease [11].

## Prospective therapeutic approaches for SCA2

To date, there is no cure for SCA2 and no therapy capable of delaying or stopping disease progression. Patient care thus relies on symptomatic and supportive treatments to counteract some symptoms. Oral supplementation with zinc together with neurorehabilitation therapy was found to promote a mild amelioration of the ataxic phenotype in SCA2 patients; however, clinical trials with a high number of patients are needed to establish the efficacy and safety of zinc supplementation [143]. Although parkinsonism is not among the most common symptoms observed in SCA2 patients, those that present that clinical manifestation respond to levodopa treatment [141, 144]. Some SCA2 patients also present myoclonus. Piracetam, a drug used for cognitive disfunction, dementia, and memory, is known to alleviate myoclonus at high doses. In an SCA2 patient with advanced pathology presenting severe myoclonus, piracetam administration significantly reduced involuntary twitching [145]. Oral supplementation with magnesium is used to prevent or reduce muscle cramps, a common symptom in SCA2 patients [144].

### Pharmacological treatments in preclinical studies

In preclinical studies, several drugs were evaluated for the treatment of SCA2. For example, chlorzoxazone, a muscular relaxant approved by the Food and Drug Administration (FDA), was shown to normalize Purkinje cells firing in a mouse model of SCA2 [146]. However, no behavioural tests were performed to assess the motor performance of the treated mice. As mentioned, calcium homeostasis is dysregulated in SCA2 patients and mouse models, where increased intracellular calcium concentration, possibly caused by mGluR1 overactivation, results in neuronal toxicity and death [23]. In line with this idea, it was shown that agonists of mGlur1 increase the responsiveness and intracellular calcium levels of Purkinje cells in SCA2 mice as compared to wild-type animals. Thus, the modulation of mGlur1 activity with selective antagonists could constitute a possible therapeutic strategy for SCA2 [147]. Accordingly, dantrolene, an intracellular calcium stabilizer, was shown to prevent Purkinje cells death and to improve motor deficits in SCA2 mouse models [23]. Recently, it was also shown that the pharmacological activation of autophagy using cordycepin mitigates SCA2-associated abnormalities [148].

### Development of advanced therapies

Because of their underlying genetic cause, SCA2 and other polyQ diseases are good targets for the development of advanced therapies based on gene and cell therapy approaches. Due to its genetic dominant character, the most straightforward approach for SCA2 would be gene silencing to prevent the translation of expanded *ATXN2*. In an SCA2 mouse model, the administration of antisense oligonucleotides (ASOs) targeting *ATXN2* mRNA led to a significant reduction of *ATXN2* mRNA and protein levels, mitigating electrophysiological abnormalities [149].

Other studies focused on gene-delivery strategies targeting several downstream molecular pathways involved in SCA2 pathogenesis. The reduction of intracellular calcium levels through viral vector-mediated expression of inositol 1,4,5-phosphatase (*Inpp5a*) resulted in an amelioration of cell dysfunctionality as well as an improvement of motor deficits in an SCA2 mouse model [150]. Recently, it was shown that G3BP1 levels are downregulated in the context of SCA2, both in patient-derived fibroblasts and in *post-mortem* brain samples [151]. To assess the functional relevance of this reduction, the study showed that the lentiviral-mediated expression of G3BP1 in a mouse model of SCA2 [148] led to a decrease in the number of mutant ATXN2 aggregates and mitigated neuronal loss [151]. Moreover, G3BP1 expression mitigated motor symptoms in a polyQ mouse model characterized by marked motor deficits [151, 152].

While the above strategies seem to be promising, when SCA2 patients display the first symptoms, it is possible that extensive neurodegeneration is already established. Thus, cell-based strategies could represent a step forward as disease-modifying therapies. Previous studies have shown that mesenchymal stem cells have neurotrophic and immunomodulatory effects [153]. Following this rationale and using an SCA2 mouse model, a study showed that the intravenous infusion of human mesenchymal stem cells led to the preservation of Purkinje cells, improved motor performance, and delayed disease onset. Nevertheless, motor improvements were mild, and observable only at one time point of the experiment. Additionally, the transplanted mesenchymal cells did not differentiate into cerebellar cells; thus, the beneficial effect observed was suggested to be due to neurotrophic factors release or cell-cell contact [154].

## Conclusion

ATXN2 is a multifaceted protein with critical roles in diverse molecular, cellular, and physiological processes. The structural features of ATXN2, including its distribution, subcellular localization, structure, and post-translational modifications, contribute to its functional versatility, ranging from RNA binding, metabolism regulation, circadian rhythm, stress granule formation, endocytosis, and cytoskeleton organization. Nevertheless, the complete picture of ATXN2 functions is still unknown, and more investigation is needed to clarify them. While the crucial biological functions of ATXN2 are uncontested, the protein is probably best known as the cause of SCA2. More than 20 years have passed since the identification of the CAG expansion mutation in the *ATXN2* gene as the cause of this disease. During this time, significant advances were made in the field, contributing to the understanding of the functional alterations promoted by the expanded ATXN2, the molecular pathogenesis of SCA2, the development of the disease manifestations, and the development of symptomatic and supportive treatments. Today, the picture that we have of SCA2 pathophysiology is more complete, and several mechanisms have been identified as being important for disease onset and progression. Clinically, a better characterization of signs and symptoms allows for a more personalized treatment, even though disease progression cannot be altered by currently available therapies. Nevertheless, there is still an urgent and unmet need for disease-modifying therapies for SCA2. Future research should focus on elucidating the interplay between various molecular pathways and identifying potential therapeutic interventions to mitigate the devastating effects of SCA2.
